# Functional and structural differences between skinned and intact muscle preparations

**DOI:** 10.1085/jgp.202112990

**Published:** 2022-01-19

**Authors:** Alex Lewalle, Kenneth S. Campbell, Stuart G. Campbell, Gregory N. Milburn, Steven A. Niederer

**Affiliations:** 1 Department of Biomedical Engineering, School of Biomedical Engineering and Imaging Sciences, King’s College London, London, UK; 2 Department of Physiology and Division of Cardiovascular Medicine, University of Kentucky, Lexington, KY; 3 Departments of Biomedical Engineering and Cellular and Molecular Physiology, Yale University, New Haven, CT

## Abstract

Myofilaments and their associated proteins, which together constitute the sarcomeres, provide the molecular-level basis for contractile function in all muscle types. In intact muscle, sarcomere-level contraction is strongly coupled to other cellular subsystems, in particular the sarcolemmal membrane. Skinned muscle preparations (where the sarcolemma has been removed or permeabilized) are an experimental system designed to probe contractile mechanisms independently of the sarcolemma. Over the last few decades, experiments performed using permeabilized preparations have been invaluable for clarifying the understanding of contractile mechanisms in both skeletal and cardiac muscle. Today, the technique is increasingly harnessed for preclinical and/or pharmacological studies that seek to understand how interventions will impact intact muscle contraction. In this context, intrinsic functional and structural differences between skinned and intact muscle pose a major interpretational challenge. This review first surveys measurements that highlight these differences in terms of the sarcomere structure, passive and active tension generation, and calcium dependence. We then highlight the main practical challenges and caveats faced by experimentalists seeking to emulate the physiological conditions of intact muscle. Gaining an awareness of these complexities is essential for putting experiments in due perspective.

## Teaser

Lewalle et al. review differences in skinned and intact muscle preparations.

## Introduction

In striated muscle, force is generated by sarcomeres located within myocytes (Bers, 2001, 2002). The sarcomere is located within the selectively permeable cell membrane, which supports intracellular ionic homeostasis. Within this highly regulated space, sarcomere force generation is activated by dynamic changes in cytosolic Ca^2+^. The sarcomeric protein troponin C (TnC) binds to Ca^2+^, which prompts the formation of myosin cross-bridges between the sarcomere thick (myosin) and thin (actin) filaments. These myofilaments are arranged in a regular lattice oriented along the muscle fiber direction and form the main structural basis of myocyte contraction. The contraction process is regulated by many other intracellular molecules and ions, in particular Mg^2+^ and H^+^, as well as by cellular and sarcomeric morphologies.

To identify the ionic and molecular mechanisms that regulate the sarcomere, it is necessary to control the chemical environment it is exposed to. The biochemistry of the sarcomere proteins can be studied using in vitro biochemistry assays. However, these fail to account for the regular structure of the sarcomere, which is important for both biochemistry and function. Alternatively, the sarcomeres can be accessed by skinning the muscle, i.e., removing the sarcolemma membrane (or making it permeable to compounds and ions), while preserving sarcomere functionality (Curtin et al., 2015). Exposing the sarcomeres to tailored ionic conditions provides a means to observe and control molecular behavior in a setting that more closely resembles native structures. After skinning, the sarcomere system is effectively isolated from the other cellular subsystems (except in some skeletal muscle experiments that remove the sarcolemma while preserving intracellular organelles and structures; Donaldson, 1985; Fill and Best, 1988; Posterino et al., 2000). This facilitates the study of contraction and its regulation separately from the sarcolemma. The central assumption of skinned muscle experiments is that the response of the sarcomeres to changes in the natural cytosol can be reproduced artificially and controllably through analogous changes in the bathing solution.

In skinning protocols (typically used with skeletal muscle) where the SR is preserved, applying caffeine liberates the intracellular Ca^2+^ reserves to stimulate contraction (Donaldson, 1985). In cases where the T tubules are preserved in the skinning process, ionic substitution in the bathing solution may induce T-tubule membrane depolarization and hence Ca^2+^ release from the SR (Fill and Best, 1988). An alternative approach to releasing SR calcium is by electric-field stimulation, with the electric field applied transversely relative to the fiber direction (Posterino et al., 2000).

The principal readouts of skinned-muscle experiments are contraction kinetics, adenosine triphosphatase (ATPase) activity, and generated force. Their value therefore rests on the premise that the structural integrity of the sarcomeres is preserved. Under this condition, skinned muscle may be viewed as an intermediary experimental system, straddling intact muscle and in vitro molecular experiments.

Skinned preparations allow the probing of muscle behavior beyond the current reach of experiments on intact systems. In experiments where contraction is elicited by controlling the bath [Ca^2+^], the influence of “cytosolic” conditions on Ca^2+^ sensitivity, in the steady-state, is typically presented in terms of Hill-type force-[Ca^2+^] relationships, or “F-pCa,” where pCa ≡ − log_10_[Ca^2+^]*/*(mol*/*liter). Other intracellular molecular structures that fulfill structural and mechanical roles (e.g., titin [Cazorla et al., 2001; Fukuda and Granzier, 2005; Fukuda et al., 2005; Li et al., 2016; Tonino et al., 2017] or the cytoskeleton [Roos and Brady, 1989]) can also be investigated. The controlled progression of the system from one equilibrium state to another has helped to reveal, for example, hysteresis in F-pCa, which may potentially fulfill a physiological role but would be difficult to identify in the dynamic natural system (Bers, 2001; Harrison et al., 1988). Dynamic mechanical experiments also yield insight into myofilament kinetics (Breithaupt et al., 2019; Palmer et al., 2020; Stelzer et al., 2006; Terui et al., 2010). In some (mechanical) skinning methods that preserve the T tubules, further details of the excitation–contraction coupling become experimentally accessible (Fill and Best, 1988; Posterino et al., 2000). The ability to perform protein-exchange manipulations (e.g., cardiac versus skeletal TnC; Babu et al., 1988; Gulati and Babu, 1989), to include fluorescent proteins (e.g., troponin; Brenner et al., 1999), and to perform time-resolved dynamics measurements through the flash photolysis of caged compounds (ATP [Goldman et al., 1982, 1984], inorganic phosphate [Araujo and Walker, 1996; Dantzig et al., 1992; Millar and Homsher, 1990; Tesi et al., 2000], and Ca^2+^ chelators [Luo et al., 2002; Wahr et al., 1998]) provide additional handles for probing molecular mechanisms. Overall, much of our understanding of striated muscle generally and cytosolic conditions (temperature, pH, etc.) is derived from skinned-muscle experiments (Bers, 2001).

Historically, skinning has been performed in a wide array of animal species and striated muscle systems, ranging from single cells to multicellular fibers of cardiac, skeletal, and smooth muscle. Various skinning techniques have been proposed. In “mechanical” skinning, the sarcolemma is effectively peeled off (entirely or partially; Cassens et al., 1986; Endo, 1977; Trube, 1978) by microdissection (Azimi et al., 2020; Donaldson, 1985; Fabiato, 1985b; Fabiato and Fabiato, 1975, 1977, 1978a, 1978b; Fill and Best, 1988; Godt, 1974; Godt and Maughan, 1977; Jewell, 1977; Lamb and Stephenson, 2018; Matsubara and Elliott, 1972; Moisescu, 1976; Rebbeck et al., 2020), while preserving the structural integrity and function of the T tubules and the SR (Lamb and Stephenson, 1990; Posterino et al., 2000; Stephenson, 1981). However, the technique is difficult and no longer used routinely. In contrast, “chemical” skinning involves dissolving or permeabilizing the membrane by applying a chemical agent. The most common agent is Triton X-100 (Solaro et al., 1971), but alternatives include Brij (Hibberd and Jewell, 1982), lubrol (Scheld et al., 1989), glycerol, and saponin (Edes et al., 1995; Endo and Iino, 1980; Gwathmey and Hajjar, 1990; Launikonis and Stephenson, 1997; Patel et al., 2001). Chemical skinning is particularly appropriate for multicellular tissue preparations. Controlling the precise protocol and chemical agent reportedly allows the selective dissolution of the sarcolemma membrane while leaving intracellular organelles (mitochondria and SR) intact. Nonetheless, treatment with (typically 1%) Triton X-100 frees the myofibrils of contamination by mitochondrial, sarcolemmal, and SR membranes while preserving ATPase activity and sensitivity to Ca^2+^ (Solaro et al., 1971). This straightforwardness makes Triton X-100 demembranation the predominantly used technique today. Other reported skinning approaches use propionate (Reuben et al., 1971) or the Ca^2+^ chelators EGTA or EDTA (Thomas, 1960; Winegard, 1971; Miller, 1979), but the uncertainty in the underlying mechanisms has undermined the reliability of these methods (Miller, 1979). For completeness, we also mention a less used “freeze drying” approach that arguably preserves the protein content of the fibers better than chemical skinning (De Beer et al., 1992; Schiereck et al., 1993; Stienen et al., 1983).

Although, for many years, skinned muscle experiments have served as an invaluable method for investigating fundamental physiology, they are increasingly inspiring more ambitious practical applications. At a practical level, live human cells are inevitably a highly scarce resource, with facilities for collecting, storing, and measuring samples often being displaced both geographically and temporally. These issues are more realistically resolved with skinned cells, which can be preserved frozen for several months (Mosqueira et al., 2019). The development of new sarcomere drugs, including omecamtiv mecarbil and mavacamten, demonstrate that the sarcomere is a viable drug target (Tsukamoto, 2019). Similarly, Ca^2+^-sensitizing drugs (which act by increasing either the sensitivity to [Ca^2+^] or the magnitude of the generated force) such as levosimendan (Edes et al., 1995), pimobendan (Fitton and Brogden, 1994; Scheld et al., 1989), sulmazole (Solaro and Rüegg, 1982), isomazole (Lues et al., 1988), and EMD-57033 (Gross et al., 1993; Lee and Allen, 1997) have all been assessed using measurements on skinned fibers. Identifying further novel sarcomere modulator compounds requires large high-throughput screening, which is unrealistic using intact muscle.

There is also a growing appetite for exploiting the quantitative value of skinned muscle experiments for more direct clinical applications, such as guiding patient-specific therapies. Much of this ambition relies on the integrative power of computational models to simulate human heart mechanics based on individual patients’ data, linking sub-cellular mechanisms with systemic behavior (Niederer et al., 2019a, 2019b). Building upon basic understanding of muscle behavior, recent developments in biomedical engineering extrapolate physiological processes at the cellular and tissue levels to predict global whole-heart function. As this field continues to grow in maturity, and as model predictions allow more meaningful comparisons with clinical data, efforts are increasingly focusing on quantitatively elucidating the interdependence between cellular behavior, tissue properties, and the anatomy. The quantitative accuracy of the subsystems at all these levels therefore becomes paramount.

In both of these evolving applications, the relevance and value of skinned-muscle experiments hinges on their ability to reliably emulate the intact system (Land et al., 2017; Margara et al., 2021; Mijailovich et al., 2021). Skinned-muscle experiments conducted over the past decades confirm the fidelity, in many respects, of these preparations as valid experimental models. However, they also highlight caveats and significant interpretational challenges. Gaining an awareness of these issues is becoming all the more essential to avoid misinterpretations that may have practical consequences. This review therefore aims to highlight these challenges, to help users of skinned-based measurements put them in an appropriate perspective.

The present review is structured as follows. We first compare measurements of the principal physiological properties of skinned and intact muscle, highlighting similarities and discrepancies. We focus primarily on chemical skinning, and in particular Triton X-100 (the predominantly used chemical agent). We then describe practical challenges involved in conducting experiments, insofar as they impact on measurement outcomes. We conclude with a summary of recommendations and main caveats.

## Comparing skinned and intact muscle

Skinned muscle experiments aim to reveal and controllably reproduce features of the physiological function of sarcomeres. However, notable discrepancies arise between skinned- and intact-muscle measurements of basic muscle properties that govern overall muscle function. To establish these differences rigorously at the single-cell level encounters significant methodological challenges. Although it might seem obvious that this would require doing measurements systematically on both preparation types in tandem, many early experiments were done predominantly on skinned rather than on intact cells (King et al., 2011). This stems largely from the specific challenges of noninjurious cell attachment and performing small-force measurement on intact single cells (Brady, 1991). More recently, technical developments (e.g., involving the use of flexible carbon fibers to hold the cells at opposite ends; Iribe et al., 2007; Le Guennec et al., 1990; Yasuda et al., 2001) have made these measurements more practicable. Despite these advances, however, only a fraction of studies in the literature have systematically made direct comparisons between skinned and intact systems taken from the same species under optimally similar conditions (see the selection listed in Table 1). Although, as discussed below, this optimization encounters many hurdles, these comparisons are essential for a reliable interpretation of the measurements.

**Table 1.** Summary of literature references of skinned-based experimental studies

Reference	System	Intact	Skinning method	[Mg^2+^] (mM)	Ionic strength (mM)	pH
Reuben et al. (1971)	Crayfish		EGTA	-	300	7.0
Winegard (1971)	Frog cardiac		EDTA	1	-	6.5–7.0
Matsubara and Elliott (1972)	Frog skeletal	X	Dissection	1	-	7.0
Godt (1974)	Frog skeletal		Dissection	5	150	7.3
Wood et al. (1975)	Human skeletal		EGTA	2–4	-	7.0
Moisescu (1976)	Frog skeletal		Dissection	1	150	7.1
Godt and Maughan (1977)	Frog skeletal	X	Dissection	3	150	7.0
Best et al. (1977)	Rat cardiac		Homogenization	0.05, 1	150	7.0
Trube (1978)	Mouse cardiac		Dissection (partial)	4	132	7.0
Gordon (1978)	Rabbit smooth		Triton X-100	1.0–6.9	130	7.0
Stienen et al. (1983)	Frog skeletal		Freeze drying	1.1	160	7.0
Fabiato and Fabiato (1975, 1978a, 1978b)	Rat cardiac		Dissection	0.32	160	7.0
Fabiato and Fabiato (1978a)	Frog skeletal		Dissection	0.32	160	7.0
Fabiato (1981)	Rat cardiac	X	EGTA	1	160	7.1
Fabiato (1981)	Rabbit cardiac	X	EGTA	1	160	7.1
Fabiato (1985b)	Canine cardiac		Dissection	3	170	7.1
Hibberd and Jewell (1982)	Rat cardiac		Brij-58	0.3	200	7.0
Solaro et al. (1971, 1976); Solaro and Rüegg (1982)	Canine cardiac		Triton X-100	Var	100	7.0
Donaldson (1985)	Rabbit skeletal		Dissection	1	150	7.0
Kentish et al. (1986)	Rat cardiac	X	Triton X-100	3	200	7.0
Fill and Best (1988)	Frog skeletal		Dissection	1	150	7.0
Lues et al. (1988)	Various cardiac		Triton X-100	-	140	6.7
Roos and Brady (1989)	Rat cardiac	X	Triton X-100	-	160	7.1
Scheld et al. (1989)	Human cardiac		Lubrol PX	-	140	6.7
Harrison and Bers (1989)	Rabbit cardiac		Triton X-100	2.2	-	7.0
Lamb and Stephenson (1990)	Toad skeletal		Dissection	1	-	7.10
Gwathmey and Hajjar (1990)	Human cardiac	X	Saponin	3	160	7.1
Sweitzer and Moss (1990)	Rat cardia, rabbit skeletal		Triton X-100	1	180	7.0
Millar and Homsher (1990)	Rabbit skeletal		EGTA	1	200	7.1
De Beer et al. (1992)	Rabbit skeletal		Freeze drying	-	-	-
Gross et al. (1993)	Guinea pig cardiac		Triton X-100	-	-	7.4
Gao et al. (1994)	Rat cardiac	X	Triton X-100	1.2	-	7.0
Wolff et al. (1995a)	Canine cardiac		Triton X-100	1	180	7.0
Edes et al. (1995)	Guinea pig cardiac		Saponin	-	160	7.4
Araujo and Walker (1996)	Rat cardiac		Triton X-100	1	180	-
Allen et al. (2000)	Rat cardiac		Triton X-100	1–8	150	7.0
Posterino et al. (2000)	Rat skeletal		Dissection	1	-	7.1
Irving et al. (2000)	Rat trabeculae	X	Triton X-100	-	200	7.35
Patel et al. (2001)	Mouse cardiac		Saponin + Triton X-100	-	180	7.0
Konhilas et al. (2002)	Rat trabeculae		Triton X-100	1	180	-
Luo et al. (2002)	Rabbit skeletal		Triton X-100	1	180	7.0
Fukuda et al. (2003)	Bovine cardiac		Triton X-100	1	180	7.0
Prado et al. (2005)	Rabbit skeletal	X	Triton X-100	-	180	7.0
Fukuda et al. (2005)	Bovine and rat cardiac		Triton X-100	1	180	7.0
Stelzer et al. (2006)	Mouse cardiac		Saponin + Triton X-100	1	180	7.0
Terui et al. (2010)	Pig cardiac		Triton X-100	1	180	7.0
Gillis and Klaiman (2011)	Fish cardiac		Triton X-100	1	170	7.0
Curtin et al. (2015)	Rabbit skeletal	X	Triton X-100	2	200	7.1
Li et al. (2016)	Rabbit skeletal		Triton X-100	-	180	7.0
Land et al. (2017)	Human cardiac		Triton X-100	1	200	7.1
Stehle (2017)	Guinea pig cardiac		Triton X-100	-	170	7.0
Breithaupt et al. (2019)	Rat cardiac		Glycerol + Triton X-100	1	200	7.0
Giles et al. (2019)	Mouse cardiac		Saponin + Triton X-100	1	180	7.0
Azimi et al. (2020)	Rat skeletal		Dissection	1	-	7.1
Rebbeck et al. (2020)	Human and rat skeletal		Dissection	1	-	7.4
Palmer et al. (2020)	Mouse cardiac		Triton X-100	1	200	7.0

A mark (X) in the Intact column indicates studies that directly compared measurements on both intact and skinned muscle (either performed within the same study or by considering previously published results). Var, variable.

### Sarcomere structure

The geometrical configuration and separation of the myofilaments regulate their interaction in the native system and hence their ability to generate tension. Under normal physiological conditions, the filament lattice structure is influenced by a complex balance of opposing forces, which include (Millman, 1998) electrostatic interactions between both thick and thin filaments (with charge being affected by pH and screened by the surrounding ionic strength), van der Waals forces, and entropic thermal forces, as well as Donnan osmotic force (whereby water enters the filament lattice to dilute counterions surrounding the charged filaments; Ilani, 2015). It is therefore unsurprising that this balance becomes disrupted upon removal of the sarcolemma.

**Figure 1.**
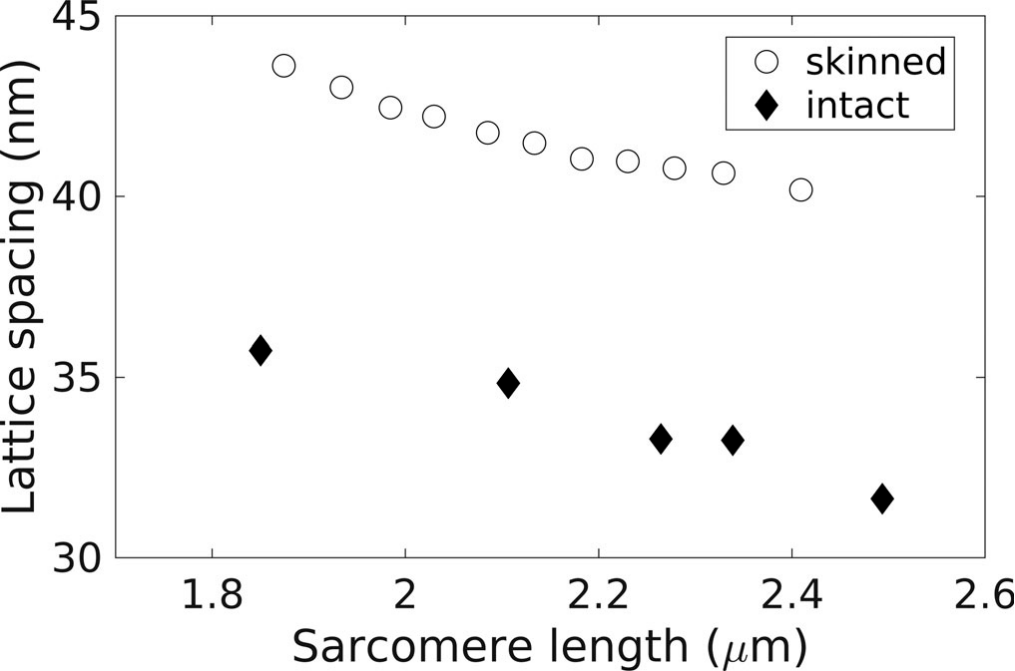
**Average myofilament spacing as a function of the sarcomere length in intact and relaxed skinned rat trabeculae, measured by x-ray diffraction.** Adapted from Irving et al. (2000).

Muscle skinning broadly conserves the sarcomere assembly, but, as illustrated below, detailed quantitative features are altered at different scales. Microscopy and synchrotron x-ray measurements on skinned muscle report a modest increase in sarcomere length (∼3%), accompanied by a greater lateral expansion (up to twofold, depending on conditions), compared with intact cells. This is apparent in both skeletal (Matsubara and Elliott, 1972) and cardiac muscle (Irving et al., 2000; Roos and Brady, 1989). In both skinned and intact preparations, longitudinal stretching decreases the myofilament lattice spacing monotonically. This occurs more slowly in the skinned system, especially at large sarcomere lengths (Fig. 1; Irving et al., 2000). Despite their similar overall behavior, different physical effects are likely to operate in the two systems. The volume of intact cells is approximately conserved (Yagi et al., 2004), and therefore, stretching the cell decreases its cross-sectional area. As the sarcomere number remains constant, this increases the sarcomere density and hence stress generation (force per unit cross-sectional area). The constant-volume constraint is removed in skinned systems (Godt and Maughan, 1977; Irving et al., 2000; Matsubara and Elliott, 1972), which allows the structure to respond more visibly to other forces.

The expansion of the myofilament spacing in skinned preparations can be reversed by increasing the osmotic pressure of the solution using dextran (Cazorla et al., 2001; Konhilas et al., 2002). However, this compressive effect does not by itself return the myofilaments fully to their intact physiological state (Konhilas et al., 2002). Recent x-ray diffraction experiments have identified an alteration of the detailed molecular structure of the thick filaments below physiological temperatures (Caremani et al., 2019, 2021). Although this effect is overlooked in many experiments, it may significantly affect cross-bridge kinetics.

Skinning may also impact sarcomere morphology on larger scales. While measuring the effect of skinning on the sarcomere length in rat heart trabeculae using laser diffraction, Kentish et al. (1986) observed an increase in the diffraction intensity and a decrease in the dispersion of the first-order diffraction. Although this effect might result from the loss of intracellular scatterers (mitochondria, cytosolic proteins, etc.) upon skinning, the authors hypothesize that the skinning process might effectively enhance the homogenization of the sarcomere environment of the skinned tissue, relative to the intact one, where individual cells may display spontaneous and uncoordinated contractions. Nonetheless, the relative homogeneity of the skinned tissue degrades rapidly after successive contractions, possibly due to a loss of integrity of the cellular structure and content, in both cardiac (Kentish et al., 1986) and skeletal muscle (Fabiato and Fabiato, 1978b). This reflects a degree of irreproducibility inherent to skinned systems.

Sarcomere structure strongly regulates contractile properties. Changes in both sarcomere length and interfilament spacing affect cross-bridge cycling and influence the regulation and amount of tension generated by skinned sarcomeres. Recent evidence also suggests that skinning may perturb myofilament interactions via steric effects due to myosin head orientations (Caremani et al., 2019, 2021; Konhilas et al., 2002). These effects, discussed further below, highlight the complexity in the disruption of the sarcomere function caused by skinning, relative to intact muscle, and the challenge in rationalizing their discrepancies based on fundamental physics principles. Ultimately, the extent to which skinning modifies sarcomere functionality bears critically on the interpretation of skinned muscle experiments.

### Passive mechanical compliance

**Figure 2.**
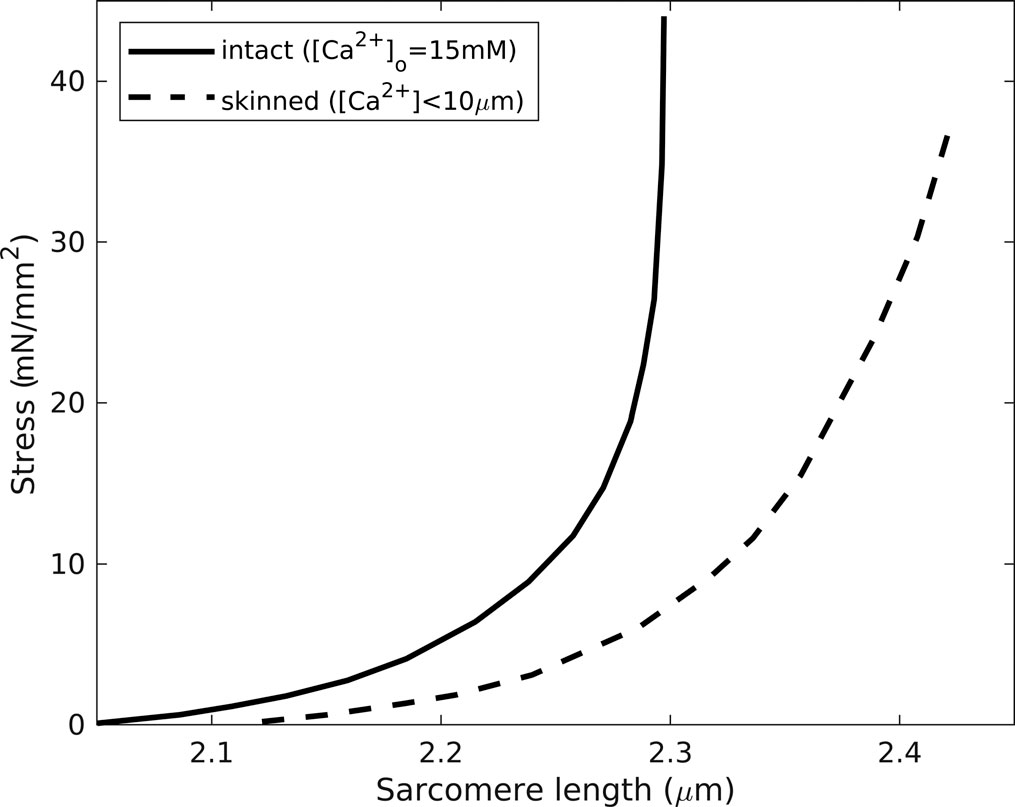
**Passive stress increasing with sarcomere length in skinned and intact rat ventricular trabeculae.** The skinned results indicate enhanced mechanical compliance. Adapted from Kentish et al. (1986). Fig. 2 is reprinted with permission from *Circulation Research*.

Passive mechanical properties of cardiac muscle strongly govern diastolic behavior. In intact tissue, these may have contributions originating in the cells themselves and the extracellular matrix (mostly comprising collagen). Passive tension and sarcomere length vary nonlinearly in both intact and skinned rat ventricular trabeculae preparations (Fig. 2; Kentish et al., 1986). However, in the skinned case, this length dependence is weaker, and the extension range is greater, indicating the presence of additional parallel elastic elements in the intact tissue, potentially associated with the sarcolemma or extracellular structures.

The qualitative similarity in the passive force-length relations in intact and skinned muscle makes the attribution of their quantitative differences challenging. The direct contribution of the sarcolemma itself, although plausible in principle, is expected to be weak, given its high compliance. However, it is more likely to contribute indirectly, given that the cell volume remains approximately constant upon stretching (Yagi et al., 2004). This effect may also be exacerbated by the Coulombic repulsion of the negatively charged myofilaments that, when confined within a fixed volume, would enhance resistance to lateral cellular compression (Kentish et al., 1986). Skinning may also cause the loss of intracellular components that contribute to the passive mechanics, e.g., a nonfilamentous stroma, comprising vesicular elements that dissolve in the skinning process (Kentish et al., 1986). Similarly, the loss of tubulin dimers from the cytoplasm may interfere with the viscoelastic behavior and resistance to cell shortening of the microtubule cytoskeleton (White, 2011).

Structural differences can also explain discrepancies between skinned and intact muscle properties. Variations in the ionic strength acting on skinned myocytes have identified a mechanical contribution from the intracellular cytoskeleton (Roos and Brady, 1989). Similarly, titin contributes to the passive stiffness in isolated myofibrils and skinned single fibers, separately from the extracellular (mostly collagen) contribution (Cazorla et al., 2001; Fukuda and Granzier, 2005; Fukuda et al., 2005; Herzog, 2018; Powers et al., 2017). Within the isolated sarcomeric system, the stiffness varies inversely with the titin molecular size (Mijailovich et al., 2019; Prado et al., 2005), but this correlation disappears in intact fiber bundles, where extracellular contributions (e.g., from collagen) may dominate (Brower et al., 2006; Chung and Granzier, 2011; Fomovsky et al., 2010).

Although the above observations highlight the limitations of using skinned preparations as a model for investigating passive mechanics in intact tissue, there may be indirect implications for contractile function. The distribution of force between passive and active mechanisms affects contraction, e.g., via force-dependent Ca^2+^ sensitivity (Cazorla et al., 2001; Fukuda and Granzier, 2005; Fukuda et al., 2005; Martyn and Gordon, 2001; Mijailovich et al., 2019; Sweitzer and Moss, 1990). In particular, passively elastic titin influences active contraction via the release of troponin I (TnI) from actin, as a result of the redistribution of mechanical load and strain on both the thick and thin filaments (Mijailovich et al., 2019). It may also determine the sarcomere length for a given afterload or the shortest sarcomere length in isotonic contractions.

### Calcium dependence of tension generation

**Figure 3.**
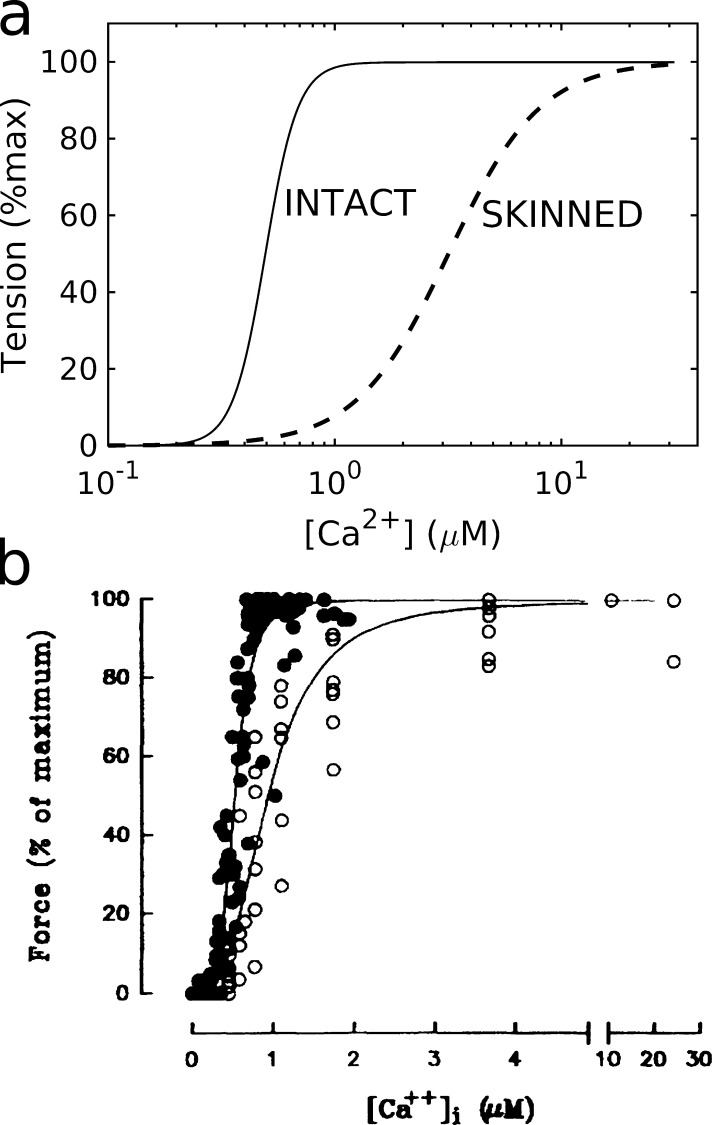
**Comparing the force-calcium relationship in intact and skinned muscle.** **(a)** Intact (ferret, 30°C; Yue et al., 1986) versus skinned (rabbit, 29°C; Harrison and Bers, 1989) muscle. **(b)** Pooled measurements derived from intact (solid symbols, pCa_50_ ≈ 6.21, *n* ≈ 4.9) and skinned (open symbols, 6.04, 3.8) preparations of the same rat ventricular myocytes. max, maximum. From Gao et al. (1994). Fig. 3 is reprinted with permission from *Circulation Research*.

Skinned preparations are often used to measure the Ca^2+^ dependence of force development under equilibrium conditions. Measured F-pCa relations (e.g., Fig. 3) are conventionally characterized by their maximum saturating value, the location of the half-maximum point (the “sensitivity,” pCa_50_), and the Hill coefficient *n* (quantifying the rate of rise and taken as a measure of cooperativity). To assess their validity, analogous F-pCa relations may also be generated in intact muscle by controlling the intracellular [Ca^2+^] homeostasis via tetanization, i.e., high-frequency activation (Fig. 3). Reported F-pCa relationships vary significantly according to the muscle type and preparations (Fabiato, 1981; Fukuda et al., 2003; Hibberd and Jewell, 1982; Kentish et al., 1986). This is problematic insofar as measurements in skinned systems aim to reproduce the “authentic” behavior in the intact system. The most intuitive mechanism involves an increased Ca^2+^-troponin binding affinity (Allen and Kentish, 1985; Kentish et al., 1986; Stephenson and Wendt, 1984), but more complex contributions also originate in the thick-filament structure upon stretching (Zhang et al., 2017).

Both pCa_50_ and *n* are significantly enhanced in the intact case (in ferret) relative to skinned tissue (rabbit), substantially exceeding typical species-dependent variability observed in skinned muscle (Fig. 3 a; Bers, 2001). A similar qualitative conclusion was drawn from comparisons of intact and skinned preparations of the same rat ventricular myocytes (Fig. 3 b; Gao et al., 1994). These discrepancies are particularly significant when comparing the measured sensitivity values (pCa_50_ = 5.52; Land et al., 2017) with physiological systolic [Ca^2+^] levels in the heart (0.6 µM ≃ pCa 6.22; Coppini et al., 2013; Land et al., 2017). Thus, the skinned muscle measurements are clearly incompatible with observed physiological behavior in intact myocytes and hence at the organ scale. Although the dominant underlying biophysical reason for these differences is uncertain, the detailed experimental conditions are fundamentally important (Bers, 2001). A rigorous quantitative comparison is therefore challenging.

Skinning may affect the F-pCa relation via the sarcomere structure. An increase in the myofilament spacing plausibly reduces the rate of myosin cross-bridge formation and hence the amount of force generated for a given [Ca^2+^]. This would translate into a reduction in pCa_50_, induced by muscle shortening, as observed in both skinned and (more weakly) intact preparations (Komukai and Kurihara, 1997). This mechanism may arguably contribute to the Frank–Starling mechanism in muscle, whereby the strength of contraction increases with stretch. However, this intuitive explanation has been shown to be insufficient in accounting for the complete effect on calcium sensitivity (Irving and Craig, 2019; de Tombe et al., 2010). It is also contradicted by experiments in which comparable myofilament spacings were achieved either via dextran-based osmotic compression or by sarcomere stretching (Konhilas et al., 2002). These discrepancies suggest that the filament spacing may not be the dominant contributor to pCa_50_. However, this conclusion assumes the functional equivalence of the two scenarios. This may not be the case, as skinning may perturb other intracellular structures (e.g., titin or thin-filament regulatory proteins; Komukai and Kurihara, 1997). Experiments on mouse skinned cardiomyocytes have suggested that titin regulates filament spacing (Cazorla et al., 2001). Osmotic pressure may also impact the cross-bridge structural configuration on smaller molecular scales (Caremani et al., 2021; Konhilas et al., 2002).

The sensitivity of the myofilaments to their chemical environment adds a further layer of complexity to skinned experiments. As discussed further below, F-pCa curves depend on the ionic strength, [Mg^2+^], and pH, all of which are routinely specified in skinned-experiment protocols. Skeletal muscle measurements have shown that increasing the temperature of the bathing solution increases the [Ca^2+^] required to activate skinned muscle as well as the maximal generated force (Godt and Lindley, 1982). Similarly, decreasing [Mg^2+^] lowers the activation [Ca^2+^] (Godt and Lindley, 1982). However, the native cell features other regulators that are lost during skinning and are not typically included in experiments. Sensitizers like taurine, carnosine-like compounds, and myosin light-chain kinase modestly increase the Ca^2+^ sensitivity (Gao et al., 1994). β-Adrenergic stimulation of intact muscle activates PKA, which in turn affects sarcomere dynamics by phosphorylating TnI and myosin-binding protein C (Gillis and Klaiman, 2011; Kentish et al., 2001; Patel et al., 2001). TnI phosphorylation decreases its binding affinity for Ca^2+^ (de Tombe and Stienen, 1995; Patel et al., 2001; Zhang et al., 1995), while that of myosin-binding protein C induces a movement of the myosin heads that accelerates force development.

Despite their appealing relative simplicity, inconsistencies between skinned and intact muscle suggest fundamental alterations to muscle function by the skinning process. Following the rapid length release and restretch of skinned rat trabeculae, force redevelopment is Ca^2+^-dependent (Wolff et al., 1995b), unlike the rate of force redevelopment after a rapid-length release of intact ferret trabeculae (Hancock et al., 1993). This discrepancy is arguably explained by the relative dominance of thin- or thick-filament kinetics, respectively (Hunter et al., 1998).

Taken together, these results illustrate the challenge of objectively determining the physiological Ca^2+^ dependence of muscle tension, in large part owing to the considerable technical challenge of replicating the native conditions of the myofilament system in vitro.

### Force-length relation

The sarcomere length dependence of force generation that underlies the Frank–Starling mechanism is a fundamental property of muscle behavior. Contributing mechanisms include the variation in myofilament overlap as the sarcomere is stretched, the apparent increase in the binding of Ca^2+^ to TnC with increasing length (Hibberd and Jewell, 1982; Kobirumaki-Shimozawa et al., 2014), and the modulation of the thick- (Fukuda et al., 2001; Zhang et al., 2017) and thin-filament structures (Zhang et al., 2017). The passive mechanical properties of titin (which vary according to the isoform) affect the variation in the lattice spacing under tension, and hence the length dependence of the actomyosin interaction (Fukuda et al., 2003). Recent evidence shows that the strain on titin, effectively acting as a force sensor, contributes to the Frank–Starling effect by influencing the structure of both the thin and thick filaments that are different from Ca^2+^-induced changes (Ait-Mou et al., 2016).

**Figure 4.**
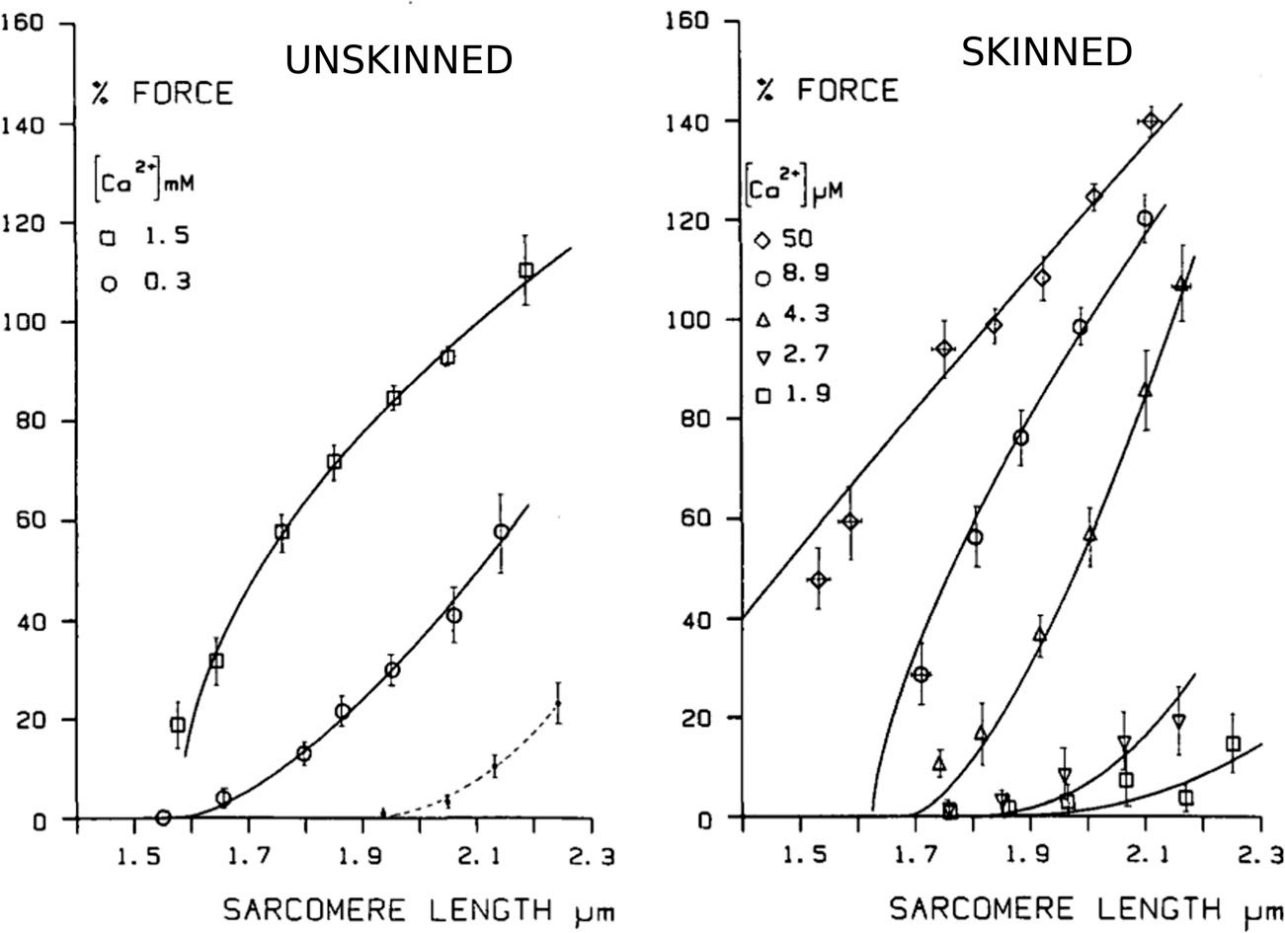
**Active force generation in intact and skinned rat ventricular trabeculae as a function of sarcomere length, for different bath [Ca^2+^].** From Kentish et al. (1986). Fig. 4 reprinted with permission from *Circulation Research*.

Length-dependent tension, manifested in the F-pCa relationship, is qualitatively similar in intact and skinned preparations (Fig. 4). In the intact case, active tension was measured as the difference between the maximum tension in transiently stimulated muscle and the resting (unstimulated) tension at the same sarcomere lengths. The process was repeated at different [Ca^2+^] values in the bathing solution, so as to modulate the intracellular calcium. Comparing Fig. 4, a and b, for sufficiently low [Ca^2+^] below the level for full activation, the skinned- and unskinned-tissue measurements show a qualitatively similar transition from a concave to a convex dependence as [Ca^2+^] is increased. The results suggest that, whereas the unskinned system sustains no active tension for sarcomere lengths below ∼1.6 µm, the skinned preparation allows tension generation in this regimen, albeit at unphysiologically large [Ca^2+^]. However, the ability to measure (potentially heterogeneous) sarcomere lengths accurately in this regimen is questionable.

For sufficiently low [Ca^2+^], the basic contraction mechanisms are thus preserved after skinning, at least qualitatively, suggesting that the general features of the force-length relationship are inherent myofibril properties. However, this conclusion assumes that (1) the chemical environments of the myofilaments are largely similar (any experimentally defined environment can only approximate the real cytosol), and (2) myofilament properties are not appreciably modified by the skinning process. The latter condition may be affected by the reported swelling of the myofilament lattice (Godt and Maughan, 1977; Irving et al., 2000; Konhilas et al., 2002; Matsubara and Elliott, 1972) or by any damage to the filaments occurring during the skinning process. Both of these effects should reduce the gradient of the tension relative to stretch.

Significant variations in measurements may originate from structural causes at different levels. The above results, derived from trabeculae, show a steeper length dependence for short sarcomere lengths, compared with those of Fabiato and Fabiato (1975) on (mechanically) skinned maximally activated single ventricular myocytes (Kentish et al., 1986). This discrepancy might be ascribed either to the conservation of intercellular connections and extracellular connective tissue that might be lost in the skinned single myocytes, or to differences in the myofilament spacing in the multicellular tissue preparation. Some more subtle effects, such as the temperature-dependent alteration of the internal thick-filament structure in demembrenated muscle, observed recently (Caremani et al., 2019, 2021), seldom receive due consideration.

**Figure 5.**
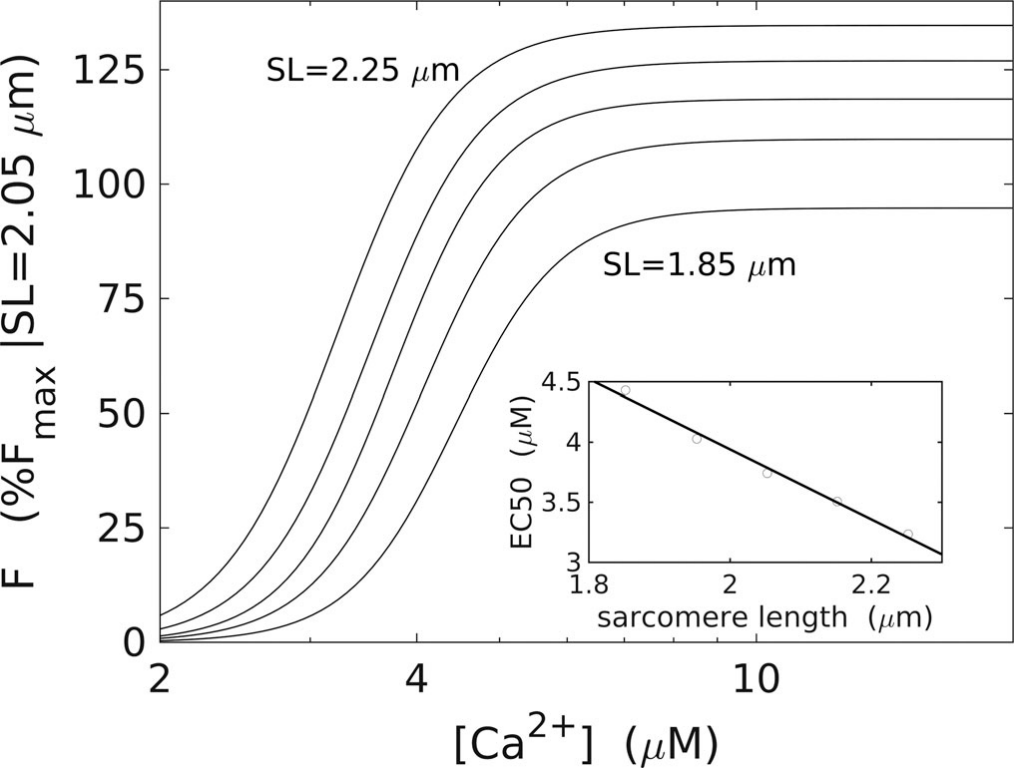
**Dependence of the calcium sensitivity on sarcomere length.** **(a)** Hill-type F-pCa for sarcomere lengths (SLs) = 1.85, 1.95, 2.05, 2.15, and 2.25 µm. Forces are normalized to the maximum force measured at SL = 2.05 µm. The data do not show a change in the Hill coefficient. **(b)** Increase in the Ca^2+^ sensitivity (decreasing [Ca^2+^] at half-maximum) with increasing SL, measured from the position of the inflection point in the fitted Hill curves from panel a. Adapted from Dobesh et al. (2002).

Length-dependent F-pCa measurements show the sensitivity of muscle activation by calcium increasing with length, as marked by an increase in pCa_50_ (Fig. 5). The maximum generated force at saturating [Ca^2+^] also increases. However, the Hill coefficient (*n* ≈ 7) does not vary significantly. A small but statistically significant increase in *n* was previously reported (Kentish et al., 1986), albeit based on sparser data, and was explained by invoking several mechanisms, e.g., interactions between adjacent tropomyosin molecules or alterations to the number of possible cross-bridges. Nonetheless, significant discrepancies even in the absolute values of *n* reported in other studies are also highlighted, potentially related to experimental conditions and the choice of skinning protocol.

The force-length relation in striated muscle underpins its central physiological role. Whereas the appeal of skinned muscle experiments for characterizing force generation is highlighted by numerous experiments, rationalizing quantitative differences remains notoriously challenging. In large part, this stems from the highly multifarious influence of the skinning process on the intracellular system and on details of the preparation protocol.

## Practical challenges: performing skinned muscle experiments

The previous section illustrated the ability of skinned muscle preparations to reproduce intact muscle behavior while highlighting significant quantitative differences between the two systems. Clarifying the sources of these differences is crucial when developing practical applications that seek to exploit skinned muscle as a reductionist model for native-state muscle. One important hurdle is to correctly replicate the chemical and physiological intracellular environment, in particular with regard to [Mg^2+^], [ATP], pH, and the ionic strength. By tuning the experimental parameters to match the physiological conditions, the consistency between skinned and intact systems can be significantly improved (Gao et al., 1994; Mijailovich et al., 2021). Over decades, systematic efforts have sought to achieve this through detailed computations of the chemical equilibria of the bathing solutions (Fabiato, 1985a; Fabiato and Fabiato, 1975, 1977; Godt and Maughan, 1977; Moisescu, 1976). In practice, experimental protocols vary, sometimes idiosyncratically, between laboratories.

This section outlines some of the elements of experimental protocols for skinned muscle that pose particular challenges insofar as they may significantly impact measurement outcomes.

### Bathing solution composition

#### ATP

After skinning, mitochondrial function is compromised, and hence, myocytes can no longer produce ATP (Rüegg, 2012). In multicellular tissue experiments, even a plentiful supply of ATP in the bathing solution may diffuse too slowly to maintain a homogeneous concentration throughout the fiber network (Godt, 1974). However, the inherent ATPase activity of muscle contraction implies a consumption of ATP supplies over the time of experiments. ATP-regenerating systems include creatine phosphate (typically 10–15 mM; Godt, 1974; Lamb and Stephenson, 2018). Nonetheless, in multicellular tissue, the rapid hydrolysis of ATP within the contractile system may yet produce an ATP concentration gradient between the interior and exterior of the network that inaccurately reflects the native state. This problem is arguably less serious in cardiac than skeletal myocytes (typical cardiac cell diameters are ∼13−20 µm, and lengths are ∼60−120 µm [Campbell et al., 1987, 1989; Liu et al., 1991], whereas skeletal muscle fiber diameters range from several microns to thousands of microns [Jimenez et al., 2013], with lengths sometimes reaching centimeters). However, the problem may yet arise in trabeculae.

The physiological role of ATP in a given experiment, in addition to its participation in cross-bridge cycling, depends on the muscle preparation. In skeletal muscle experiments that preserve intracellular membrane structures (Endo and Iino, 1980; Launikonis and Stephenson, 1997), ATP governs calcium pumping into the SR (Godt, 1974; Lamb and Stephenson, 2018). This function is of course nonexistent in preparations where the SR has been dissolved. Alongside its role as energetic fuel, ATP also maintains the extensibility of the muscle by allowing myosin to dissociate from actin (Best et al., 1977; Weber and Murray, 1973).

**Figure 6.**
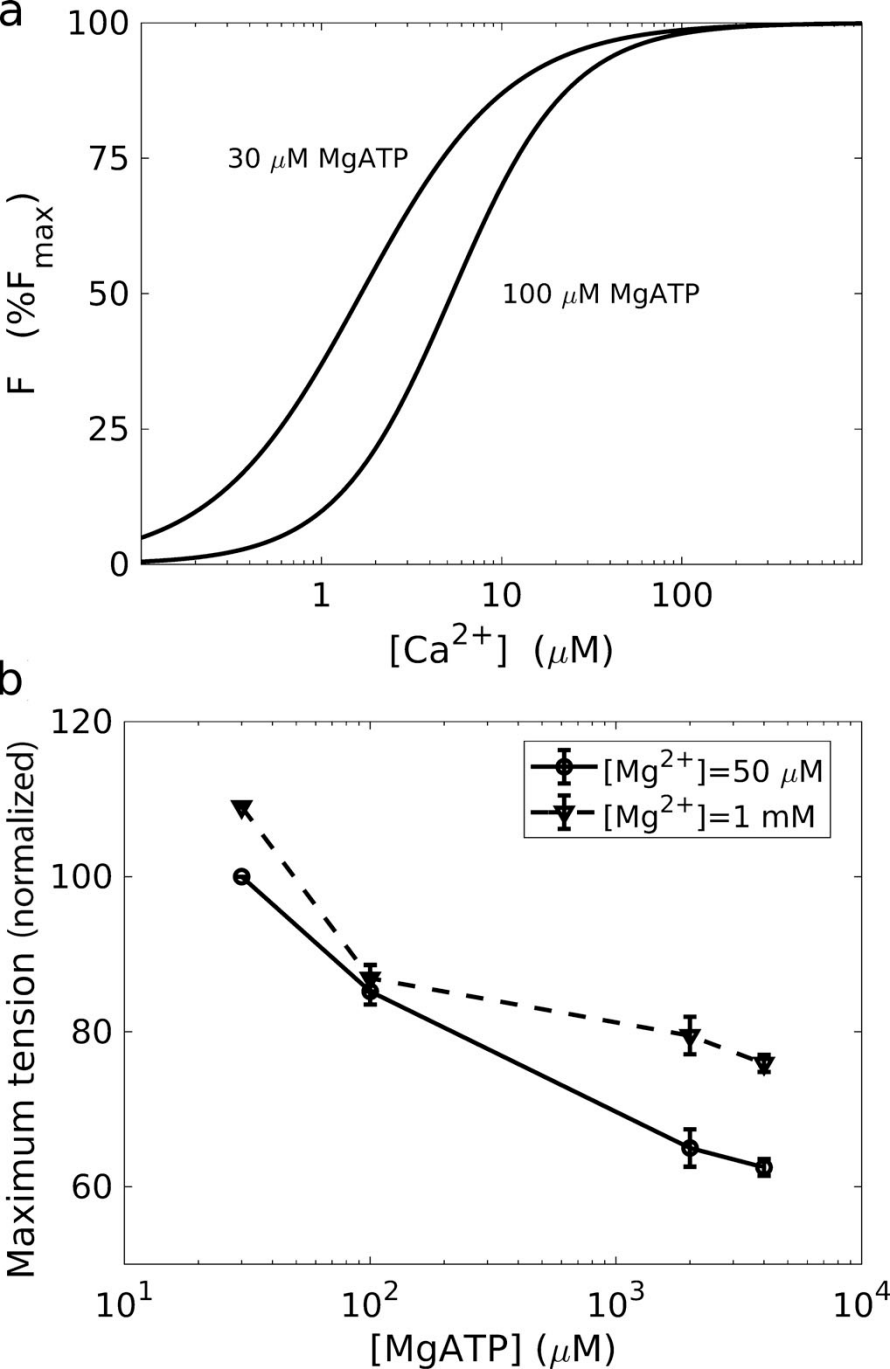
**Dependence of the force–calcium relationship on MgATP in the rat heart.** **(a)** Decrease in Ca^2+^ sensitivity (increase in [Ca^2+^] at half-maximum) as [MgATP] increases from 30 to 100 µM ([Mg^2+^] = 50 µM). **(b)** Decrease in the maximum tension with increasing [MgATP]. Adapted from Best et al. (1977).

The decrease in maximum force with increasing [ATP] (in its physiological form MgATP; Fig. 6 b) is intuitively explained by the reduction in the number of formed cross-bridges (since ATP binding is associated with the release of rigor myosin; Best et al., 1977). An accompanying decrease in pCa_50_ and an increase in the Hill coefficient (Fig. 6 a; Best et al., 1977) are both complicated by their Mg^2+^ dependence. These observations have been explained in terms of the effective cooperativity between neighboring cross-bridges in altering the inhibitory properties of troponin, which would arguably increase cross-bridge activation at a given [Ca^2+^] (Best, 1983; Best et al., 1977; Weber and Murray, 1973). However, this scenario is difficult to reconcile with analogous studies in skeletal muscle that report a qualitatively similar behavior for pCa_50_ but with little [MgATP] dependence on maximum tension (Godt, 1974).

#### Mg^2+^

Mg^2+^, the second most abundant cation in muscle cells after K^+^, regulates the Ca^2+^ sensitivity of myofilament activity via its binding affinity to troponin (Alpert et al., 1979; Bers, 2001; Best, 1983; Best et al., 1977; Rayani et al., 2018; Tikunova and Davis, 2004). The Ca^2+^-specific low-affinity binding site (site II) at the N-terminal end of cardiac TnC serves as the principal initiator of contraction in the presence of Ca^2+^ (Bers, 2001). However, the structure of TnC is also controlled by binding sites III and IV, located at the C-terminal end, which competitively bind either Ca^2+^ (with high affinity) or Mg^2+^ (low affinity; Rayani et al., 2018; Tikunova and Davis, 2004). According to some cardiac muscle experiments, more Ca^2+^ is required to achieve a given degree of activation as [Mg^2+^] increases in the millimolar range (Best, 1983; Tikunova and Davis, 2004), consistent with competitive binding of these ions on TnC. However, this interpretation is contested by other cardiac experiments claiming negligible impact to the Ca^2+^ sensitivity under even an order-of-magnitude change in Mg^2+^ (Allen et al., 2000). The precise effect of Mg^2+^, while being potentially artifactual in some cases, may also vary with the dominant mechanism of action in the specific muscle system considered.

Historically, setting the physiologically correct [Mg^2+^] has been challenging. Its determination requires the consideration of multiple binding equilibria and is naturally prone to uncertainty (Lamb and Stephenson, 2018). Given its relative abundance, cytosolic Mg^2+^ was initially assumed to merely ensure the balance for anionic charge, but its regulatory role was recognized subsequently. Various techniques have measured [Mg^2+^] (using spectrophotometry, Mg^2+^-sensitive electrodes, dye-based measurements, etc.). However, these measurements carry significant uncertainties, particularly given the difficulty of discerning free cytosolic Mg^2+^ from the total cellular magnesium (up to 20 times greater, contained in MgATP or cellular compartments) or interference from other ions (Romani and Scarpa, 1992). Many measurements report [Mg^2+^] as being consistently 0.4–0.8 mM but reaching up to 3.5 mM in some cases (Romani and Scarpa, 1992). In the intact rat heart specifically, values of 0.72 mM (from epifluorescence; Gao et al., 1994) or 0.85 mM (^19^F-NMR; Murphy et al., 1989) have been measured. [Mg^2+^] in excess of several millimolars are used in some studies but are known to be above the physiological level (Bers, 2001; Hunter et al., 1998).

#### pH

Intracellular pH in intact muscle regulates all the stages of tension generation, including the handling of Ca^2+^ by sarcolemmal electrophysiology, its delivery to the myofilaments, and the response of the filaments to the Ca^2+^ signal (Orchard and Kentish, 1990). This versatility makes it difficult to establish the relative significance of pH on sarcomere function specifically.

**Figure 7.**
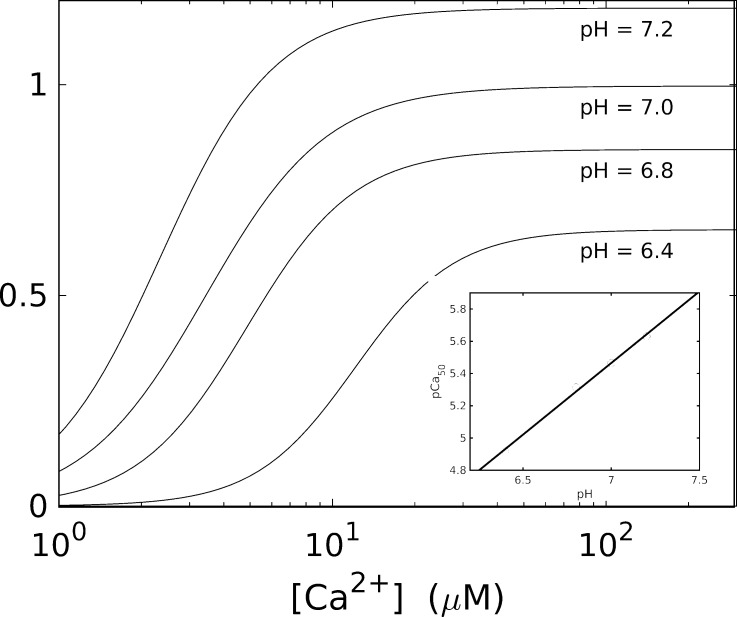
**Dependence of pH on the force-calcium relationship in guinea pig trabeculae.** Adapted from Orchard and Kentish (1990).

In skinned muscle, a decrease in pH decreases pCa_50_. The results in Fig. 7 show a 0.1% drop in pH producing a 0.1% drop in pCa_50_ (Bers, 2001; Orchard and Kentish, 1990). The precise mechanism for this effect remains uncertain but may involve competition of H^+^ with Ca^2+^ for binding to TnC, interactions within the troponin complex, or the shielding of the net effective negative charge of the TnC binding site (Orchard and Kentish, 1990). Although a decrease in calcium sensitivity was also confirmed qualitatively in tetanized intact cardiac muscle (Marban and Kusuoka, 1987), the results differ quantitatively.

The observed decrease in maximal force resulting from decreasing pH in skinned muscle may be due to a direct impact on the efficiency of the coupling of ATP hydrolysis to cross-bridge force generation (Fig. 7; Orchard and Kentish, 1990). ATPase activity is affected by pH in intact muscle, albeit more weakly (Blanchard and Solaro, 1984; Kentish and Nayler, 1979; Orchard and Kentish, 1990). However, it is uncertain whether the same dominant mechanisms are relevant in the intact and skinned cases.

The suitability of skinned muscle experiments for reliably investigating pH dependence is thus questionable. Bathing solutions for skinned muscle are typically designed with a high pH-buffering capacity (e.g., with 90 mM HEPES) to maintain a stable pH ∼7 (see Table 1) when large proton fluxes are generated during ATP hydrolysis (Lamb and Stephenson, 2018).

#### Ionic strength

**Figure 8.**
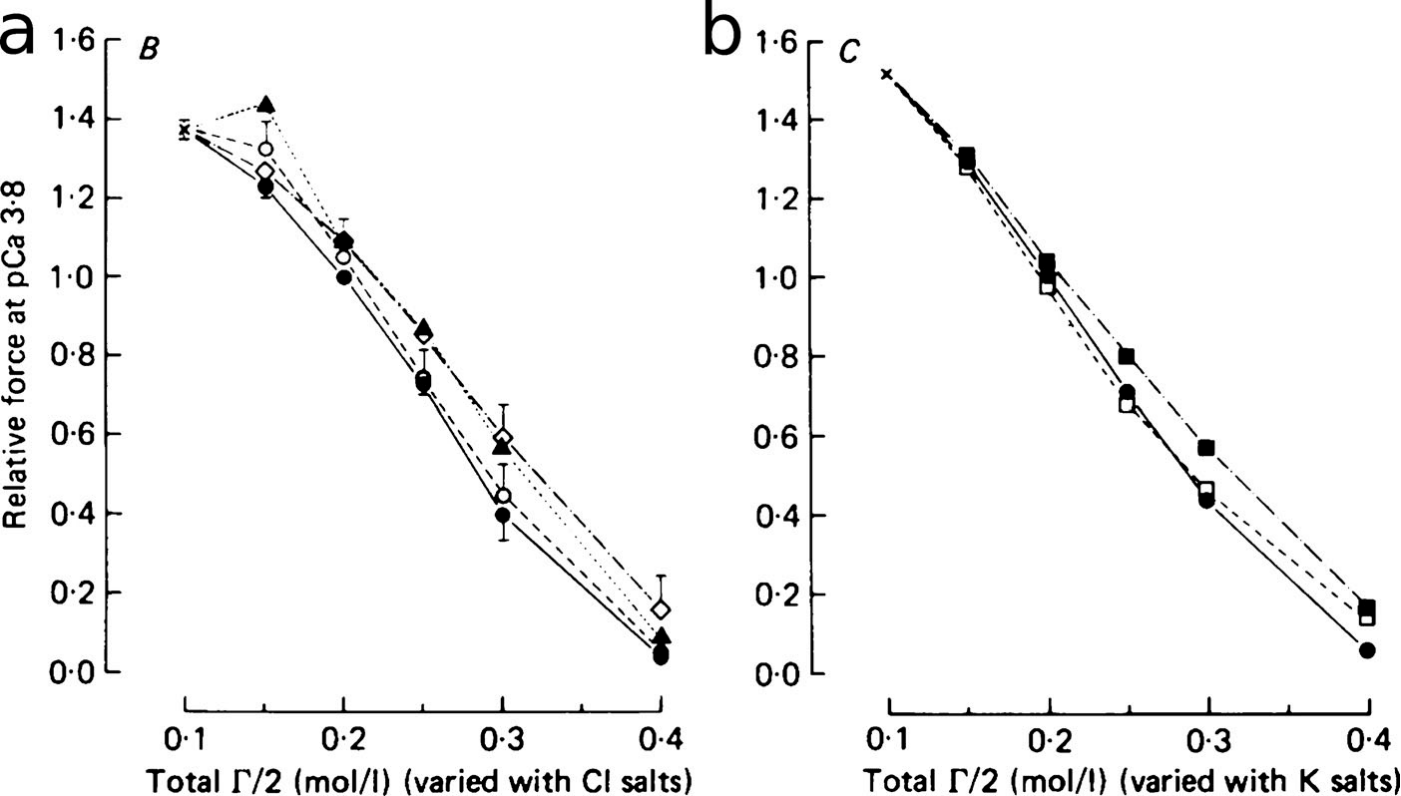
**Dependence of generated tension on osmolarity.** The osmolarity Γ*/*2 was controlled by varying (a) the Cl*^−^* salt (filled circles: KCl; open circles: NaCl; diamonds: TMACl; triangles: choline Cl) or (b) K^+^ salt concentrations (filled circles: KCl, filled squares: K propionate; open square: K Mes), for pCa = 3.8. The consistency between the results suggests that the tension depends predominantly on the ionic strength rather than on the size of specific ions. From Kentish (1984). Fig. 8 reprinted with permission from *Journal of Physiology*.

Ionic strength impacts inversely on the maximum force generated by skinned muscle (Fig. 8; Kentish, 1984). In practice, it can be controlled experimentally, in both cardiac and skeletal experiments, for example by varying KCl in the bathing soution (Kentish, 1984; Solaro et al., 1976). Reported ionic strength values range between 150 and 200 mM (Table 1), reflecting the difficulty of assigning a correct value objectively. The relatively weaker changes in force produced by varying osmolarity independently (e.g., by the addition of sucrose) or by substituting specific ionic species suggest that this effect is related to the charge of the solutes rather than the osmolarity of the solution per se or to the inhibitory effect of particular ions (Fig. 8). The inhibition of tension appears to be associated with Ca^2+^ binding, as this ionic strength dependence is [Ca^2+^] dependent only in the presence of MgATP (in skeletal muscle; Solaro et al., 1976). However, the precise ionic strength in intact muscle is uncertain (Gao et al., 1994), as reflected in the lack of consensus in the literature (see Table 1).

#### Conclusion

The above considerations of ATP, Mg^2+^, pH, and ionic strength highlight the sensitivity of skinned muscle measurements to the precise solution composition. Establishing the correct recipe is made all the more challenging given that the impact on measured force generation varies between muscle systems and species. As argued above, although differences between measurements often appear to be quantitative, this does not exclude the possibility of qualitative differences in the dominant mechanisms of action. This fundamental ambiguity introduces considerable complication in translating results meaningfully to the intact system.

### Temperature

Physiological function emerges from the balance of multiple temperature-dependent processes. Although measurements should thus ideally always be done at physiological temperature, lower temperatures are often used in practice due to the impaired stability of the sarcomere structure in skinned preparations at higher temperatures. This can have significant consequences on contraction, given the highly variable temperature sensitivities of different subcellular mechanisms (Rall and Woledge, 1990).

**Figure 9.**
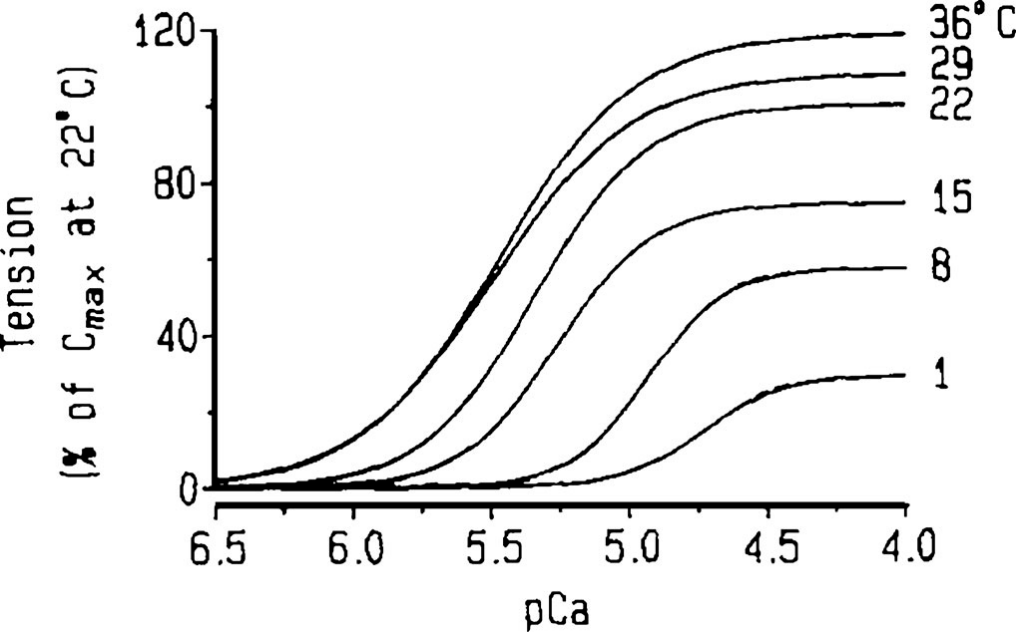
**Temperature dependence of the F-pCa relationship in skinned trabeculae from the rabbit ventricle, showing an increase in both the maximum tension C_max_ and the sensitivity pCa_50_ (pCa at half**-**maximum) with increasing temperature.** Adapted from Harrison and Bers (1989).

There is widespread agreement that cooling reduces the maximum generated force in a wide range of muscle types and preparations (Fig. 9; Fabiato, 1985b; Godt and Lindley, 1982; Harrison and Bers, 1989; Stephenson and Williams, 1985; Sweitzer and Moss, 1990). This result has been argued to result more from a change in the force exerted by cross-bridges than from the number of cross-bridges formed (Sweitzer and Moss, 1990). In contrast, the temperature dependence of calcium sensitivity is less consistent. Skinned muscle displays either an increase (Brandt and Hibberd, 1976; Harrison and Bers, 1989; Orentlicher et al., 1977; Sweitzer and Moss, 1990) or a decrease in pCa_50_ (Fabiato, 1985b; Godt and Lindley, 1982; Stephenson and Williams, 1985) with increasing temperature. However, the former result may be an artifact associated with heterogeneous shortening of sarcomeres at higher temperatures (Sweitzer and Moss, 1990).

More recent work has revealed further complications in the regulatory role of temperature in muscle. In particular, temperature influences structural thick-filament regulation in both cardiac and skeletal muscle (Caremani et al., 2019, 2021; Park-Holohan et al., 2021). Reducing the temperature disrupts the orderly configuration of the myosin lever arms along the thick filaments, making them less available for force generation and causing an almost threefold decrease in total tissue force.

The above experimental results highlight the multifaceted complexity of temperature dependence that arises from the interdependence of multiple molecular processes. Skinned preparations constitute only a subsystem within the overall muscle system, and there is therefore no guarantee that the kinetic balance within the reduced system is physiologically accurate.

### Sarcomere heterogeneity

For conceptual convenience, muscle tissue is often represented as a homogeneous assembly of identical sarcomeres acting in synchrony. This picture is simplistic in reality. Aspects of muscle dynamics, even under isometric conditions, derive specifically from the heterogeneous behavior at the sarcomere level. For example, within a myofibril, tension relaxation proceeds with the onset of rapid lengthening (“give”), initially in a single weak sarcomere, that then propagates to other sarcomeres along the myofibril (Edman and Flitney, 1982; Poggesi et al., 2005; Stehle, 2017). This effect accounts for the [P_i_]-dependent asymmetry in the force kinetics that is observed in contraction-relaxation cycles when [Ca^2+^] is stepped up and down (Poggesi et al., 2005). It also suggests that relaxation kinetics is governed not only by the rate-limiting steps of the cross-bridge cycle of a generic myosin molecule but also by collective effects at a higher structural level.

This effect arguably escapes notice in skinned-fiber experiments that exploit the flash photolysis of caged compounds to time-resolve the details of cross-bridge–cycle kinetics (e.g., the photorelease of inorganic phosphate P_i_ modulates cross-bridge kinetics; Araujo and Walker, 1996; Dantzig et al., 1992; Millar and Homsher, 1990; Tesi et al., 2000). These experiments suffer from important practical limitations. In particular, the relatively modest (unidirectional) changes in [P_i_] achievable by photorelease fail to disrupt the chemomechanical equilibrium of the sarcomeres sufficiently to generate heterogeneous give. Under these near-equilibrium conditions, observed changes in force are more likely to reflect rate-limiting single-cross-bridge kinetics than transients in sarcomere heterogeneity. This obstacle was bypassed in experiments done on isolated myofibrils, which, in contrast, allow sufficiently large jumps in [P_i_] (in both directions) to be imposed by rapid solution change (Poggesi et al., 2005; Stehle, 2017). By monitoring the progression of tension decay in conjunction with the lengths of individual sarcomeres, these experiments highlight the role of sarcomere dynamics in accounting for tension relaxation. Compared with skinned-tissue experiments, they also provide better consistency with the relaxation kinetics (*k*_TR_) observed in mechanically induced force redevelopment (Stehle, 2017).

## Practical considerations

The preceding discussion has highlighted the value of skinned muscle in emulating the essential features of intact muscle contraction in vivo. On the other hand, we have also described how discrepancies between intact and skinned muscle properties are sufficiently significant as to mar the prospect of considering skinned preparations as unambiguous surrogates. The underlying causes are complex, and it is often difficult to distinguish between experimental artifacts and manifestations of genuine physiological differences. This complexity is further compounded by species- or system-dependent specificities (e.g., cardiac versus skeletal muscle). Consequently, in practice, experimental protocols often evolve organically within laboratory communities, based on direct observations and acquired practical knowhow. Interestingly, a recent meta-analysis of published measurements of specific force in skinned human skeletal muscle noted a greater consistency in the results obtained within research groups (defined in terms of commonalities in authorship) than between them (Kalakoutis et al., 2021). This observation could be interpreted as revealing a genealogy of sorts in the evolution of protocols that is at odds with rigorous and objective development, thereby possibly mitigating the appeal of the experiments altogether.

Tempting as it may be to imagine a universally applicable method, we feel it would be counterproductive to seek to disentangle and confront the rationales of individual protocols, with the risk of dogmatically promoting one valid method among several. The very idea of a unique universal recipe, valid for all experiments, is indeed highly questionable. As a more fruitful approach, we instead present the following themes as set of general guiding principles for encouraging good experimental practice.

### Monitoring sarcomeric dynamics

Given the importance of sarcomere length and interfilament dynamics in force generation, we recommend that mechanical force measurements be accompanied by the simultaneous measurement of striation patterns. This would include the mean sarcomere length and, ideally, an index of heterogeneity and/or stability. We recognize that these measurements may be particularly challenging in cardiac trabeculae.

### Fixing the pH

Ensuring the constancy of pH is paramount for ensuring consistency in measurements. This is achieved by applying a suitable buffer, in many cases imidazole.

### Saturation with ATP

A useful simplification of the experimental system is to ensure that the cross-bridge cycling kinetics is not rate-limited by ATP. In most cases, this can be achieved by using solutions with at least 4 mM free ATP.

### Careful control of [Ca^2+^]

The importance of correctly determining the concentration of free Ca^2+^ cannot be sufficiently emphasized. Some laboratories use pCa solutions based on recipes that originate with Fabiato and Fabiato (1979) or Godt and Lindley (1982). Those wishing to make new recipes can consider using the MaxChelator software suite (Bers et al., 2010; Patton et al., 2004), which can provide appropriate stoichiometric concentrations of Ca^2+^, Mg^2+^, EGTA, and ATP for use in experimental solutions. A useful recipe for producing buffers with varying [Ca^2+^] is to prepare “low” and “high” reference buffers (e.g., with pCa = 9.0 and 4.5) and to mix them in appropriate proportions.

### Choice of temperature

Given the importance of temperature as a determinant of muscle kinetics, it stands to reason that experiments should be done at physiological temperatures. However, a practical drawback is its destabilization of the sarcomere structure. Skeletal fibers have historically been measured at lower temperatures (sometimes even near above freezing) to ensure that preparations last the experiment duration. Many experiments on both skeletal and cardiac muscle can be done at 15°C. However, it is worth noting that rodent myocardium is more fragile than human (where room temperature or even 37°C is possible), possibly owing to differences in metabolic and ATPase rates. As a general recommendation, we would encourage experimentalists to choose temperatures that are nearest to physiological conditions where the preparation is stable. It is, however, perhaps even more important to only compare experimental results obtained at the same temperature.

## Conclusion

The aim of this review was to survey the benefits of skinned muscle measurements for characterizing cardiac muscle physiology, while highlighting intrinsic challenges for both the conduct and the interpretation of measurements. These features are summarized in Table 2. Apparently quantitative differences between skinned and intact muscle measurements (often dependent on species, muscle type, or sample preparation) may in fact stem from qualitative differences in dominant molecular mechanisms, which are often difficult to discern with certainty. The current ambition to expand the scope and practical applications of these experiments is making such challenges all the more noteworthy.

**Table 2.** Summary of strengths and weaknesses of skinned muscle experiments

Strengths
• Direct access to the sarcomere system
• Separation of cellular subsystems (e.g., sarcomeres versus sarcolemma)
• Ability to use fluorescent probes and other analytic tools
• Convenience of controllably performing different standardized experiments (e.g., isometric/isotonic contractions)
• Ability to perform protein exchange experiments that preserve overall functionality (e.g., troponin; Babu et al., 1988; Brenner et al., 1999; Gulati and Babu, 1989); and to probe time-resolve sarcomere dynamics by photolysis of caged compounds (ATP [Goldman et al., 1982, 1984], inorganic phosphate [Araujo and Walker, 1996; Dantzig et al., 1992; Millar and Homsher, 1990; Tesi et al.*,* 2000], and Ca^2+^ chelators [Luo et al., 2002; Wahr et al., 1998])
• Simpler handling and storage logistics (samples can be thawed and analyzed after prior freezing)
**Weaknesses**
• Challenge of reproducing the native physiological environment
• Variations in results between laboratories
• Instability and sensitivity to temperature
• Challenges of [Ca^2+^] calibration
• Structural changes caused by skinning (e.g., altered sarcomere morphology, loss of cellular heterogeneity), impacting functional behavior

The potential pitfalls of mischaracterizing sarcomere behavior, based on skinned muscle measurements, are particularly exposed when considering the broader physiological context, where different cardiac subsystems operate simultaneously (Mosqueira et al., 2019; Niederer et al., 2019b). Pharmacological research increasingly exploits skinned muscle experiments to assess targeted drug action on sarcomeres (Dou et al., 2007; Edes et al., 1995; Fitton and Brogden, 1994; Hara et al., 1999; Kobayashi et al., 1991; Lamont and Miller, 1992; Lee and Allen, 1997; Lues et al., 1988; Scheld et al., 1989; Solaro and Rüegg, 1982; Sudo et al., 2001; Tadano et al., 2010). However, drug impact is notoriously multifaceted, and side effects, unseen in the isolated sarcomeres, may readily and unpredictably overwhelm intended effects (Lee and Allen, 1997; Lues et al., 1993). These side effects notwithstanding, the extrapolation of skinned-muscle measurements to the native cellular state and to systemic cardiac function encounters significant interpretational hurdles, as illustrated above.

Skinned muscle measurements carry intrinsic uncertainty, as experiments performed using different animal models, temperatures, and protocols occasionally produce contradictory characterizations. Approximate quantitative accuracy is obviously highly problematic in the perspective of developing customized clinical care. This requirement is particularly important given the modular nature of models and the need to combine interacting subsystems on different length scales (Niederer et al., 2019a, 2019b). In practice, the interfacing of such modules normally requires ad hoc empirical alterations to model parameters, often relying on the modeler’s judgment (Hunter et al., 1998; Land et al., 2017). These choices are naturally often speculative.

Despite these difficulties, it would be wrong to misrepresent the true potential of skinned-muscle experiments. Just as animal models are essential for investigating human physiology, skinned muscle provides an experimental setting with unique benefits. Biophysical modeling helps to formalize the conceptual basis for interpreting experimental data in terms of specific mechanisms (for example, an observed variation in pCa_50_ may result from changes to troponin binding kinetics or cross-bridge formation). Global sensitivity analyses allow a ranking of the relative importance of individual model parameters, thus providing a handle for guiding judgment in how to use measurement-derived parameters (Longobardi et al., 2020). In this perspective, the benefit of models is in providing a framework for formulating and testing hypotheses, rather than delivering fixed and absolute representations of the muscle system.

The appeal of skinned muscle preparations is best appreciated by seeing them not as a direct emulation of real muscle, but rather as one further element in the physiologist’s experimental armory. This issue is well illustrated by Irving and Craig (2019) with reference to a loosening of the thick-filament structure induced by cardiac myosin-binding protein C phosphorylation. This effect was manifested as a structural change in skinned cardiac muscle but may be eclipsed in the compact and crowded conditions of intact muscle. In such circumstances, attempting to reconcile the experiments, even qualitatively, may seem futile. Yet the skinned-muscle effect may well be the telltale indicator of a genuine regulatory mechanism that would otherwise remain invisible and unmeasurable in the intact system. Rather than seeking a literal mirroring of these skinned and intact experiments at any cost, additional physiological insight might potentially be gained by further pursuing the experiments, and comparing their quantitative results in parallel, in other cell types or under different experimental conditions. Ultimately, the integration of experimental findings remains a continual process involving a balance of pragmaticism and biophysically guided scientific judgment.
